# Lupus cardiomyopathy: a reversible form of left ventricular dysfunction

**DOI:** 10.1186/ar3995

**Published:** 2012-09-27

**Authors:** ML Ishimori, M Agarwal, RK Ng, LD Nugent, DJ Wallace, RJ Siegel, MH Weisman

**Affiliations:** 1 Cedars Sinai Medical Center, Los Angeles, CA, USA

## Background

Myocarditis has been reported to be a common postmortem finding of systemic lupus erythematosus (SLE) patients. However, most case reports on SLE cardiomyopathy have not found myocarditis on biopsy. Stress-related cardiomyopathies result in reversible left ventricular (LV) dysfunction. The purpose of this study is to characterize the nature and course of LV dysfunction in SLE patients.

## Methods

We evaluated a large cohort of SLE patients hospitalized at Cedars Sinai Medical Center between 28 August 2001 and 30 October 2010. Patients were included in the study that met American College of Rheumatology criteria for SLE, had an erythrocyte sedimentation rate and high sensitivity C-reactive protein performed, and had an echocardiogram with ejection fraction (EF) <45% during index hospitalization. Admission data, medications, and echocardiograms were reviewed.

## Results

Five-hundred and twenty-six SLE patients were surveyed, of which 15 patients met all study inclusion criteria with LVEF ranging from 15 to 45%, mean 33 ± 9.8%, Twelve of 15 patients demonstrated a reversal of acute cardiomyopathy, showing an improvement in LVEF from 10 to 40%, mean 23.4 ± 9%. Twelve patients had generalized LV hypokinesis. Two patients underwent coronary angiography and had no obstructive coronary lesions. One patient also underwent cardiac biopsy, which did not show any evidence of myocarditis. Of the three patients whose cardiomyopathy did not reverse, all died due to their underlying medical illness.

## Conclusion

This is the first report to describe a reversible cardiomyopathy in SLE patients. The pattern of wall motion abnormalities and its reversibility is more indicative of a stress-related cardiomyopathy syndrome than being the result of myocarditis.

